# Inhibition of murine hepatoma tumor growth by cryptotanshinone involves TLR7-dependent activation of macrophages and induction of adaptive antitumor immune defenses

**DOI:** 10.1007/s00262-019-02338-4

**Published:** 2019-06-03

**Authors:** Zhen Han, Shuo Liu, Hongsheng Lin, Anna L. Trivett, Sean Hannifin, De Yang, Joost J. Oppenheim

**Affiliations:** 1 Cancer and Inflammation Program, Center for Cancer Research, National Cancer Institute, Frederick National Laboratory for Cancer Research (FNLCR), Rm 21-89/31-19, Bldg 560, 1050 Boyles Street, Frederick, MD 21702-1201 USA; 2 Guang’ Anmen Hospital, China Academy of Chinese Medical Sciences, #5 Beixian Ge, Xi Cheng District, Beijing, 100053 China

**Keywords:** Cryptotanshinone, Hepatoma, Apoptosis, STAT3, Macrophage, Anti-PD-L1

## Abstract

Cryptotanshinone (CT), a purified compound initially isolated from the dried roots of *Salvia militorrhiza.* Bunge, exhibits cytotoxic antitumor effects on many tumors. We have shown that CT possesses the dual capacities to concomitantly inhibit the proliferation of lung cancer cells and promote the generation of antitumor immunity. In this study, we investigated whether CT could be used to treat hepatocellular carcinoma (HCC) using a mouse Hepa1-6 model. CT inhibited the proliferation of mouse hepatoma (Hepa1-6) cells in vitro by inducing Hepa1-6 cells apoptosis through the JAK2/STAT3 signaling pathway. In addition, CT activated macrophages and polarized mouse bone marrow-derived macrophages (BMM) toward an M1 phenotype in vitro, which depended on the TLR7/MyD88/NF-κB signaling pathway. Furthermore, CT significantly inhibited the growth of syngeneic Hepa1-6 hepatoma tumors, and, in combination with anti-PD-L1 cured Hepa1-6-bearing mice with the induction of long-term anti-Hepa1-6 specific immunity. Immunoprofiling of treated Hepa1-6-bearing mice revealed that CT-promoted activation of tumor-infiltrating macrophages and dendritic cells, induction of antitumor T cell response, and infiltration of effector/memory CD8 T cells in the tumor tissue. Importantly, the immunotherapeutic effects of CT and anti-PD-L1 depended on the presence of CD8 T cells. Thus, CT and anti-PD-L1 may provide an effective immunotherapeutic regimen for human HCC based on a combination of cytotoxic effects and induction of tumor-specific immunity.

**Electronic supplementary material:**

The online version of this article (10.1007/s00262-019-02338-4) contains supplementary material, which is available to authorized users.

## Introduction

The antitumor effects of checkpoint inhibitors have revealed the importance of immune-mediated antitumor defenses [1]. Macrophages are crucial contributors to innate immunity and also function as antigen-presenting cells (APCs). However, macrophages are heterogeneous and can exert opposing functions that promote tumor development and progression [2]. In particular, tumor associated macrophages (TAMs) displaying the M2 phenotype promote tumor growth and metastasis. In contrast, the M1 counterpart possesses proinflammatory and tumor suppressive properties [2, 3]. Macrophages display plasticity and change their functional profiles in response to environmental stimuli. Thus, the reprogramming of macrophages toward M1 phenotype is believed to be a key target of antitumor immunotherapy [4].

Cryptotanshinone (CT) is a well characterized compound derived from traditional Chinese medical (TCM). CT, initially extracted from the root of *Salvia miltiorrhiza.* Bunge, is one of several tanshinone derivatives, including tanshinone I, IIA, and IIB and dihydrotanshinone [5]. More recently, CT has been purified, synthesized, and biochemically characterized. Many researchers are currently investigating CT and have reported that CT exhibits direct cytotoxic effects on multiple types of cancer cells [6–11]. We have recently demonstrated that CT exhibits dual antiproliferative effects on mouse Lewis lung carcinoma (LLC) cells as well as a dendritic cell (DC)-maturing effect (see accompanying paper by Liu et al., Cancer Immunol Immunother 2019), [10.1007/s00262-019-02326-8]. CT inhibits LLC proliferation by activating p53, downregulating cyclin B1 and Cdc2, and consequently resulting in G2/M cell-cycle arrest. In addition, CT promoted DC maturation, as evidenced by upregulation of costimulatory and MHC molecules, and elevated production of proinflammatory cytokines (e.g., TNFα, IL-1β, and IL-12p70), using a signaling pathway that relies on the presence of MyD88.

Immunotherapy of cancers with checkpoint inhibitor blocking antibodies, such as anti-PD-L1 or anti-CTLA4, is only effective for about ¼ of patients with preexisting tumor-infiltrating effector T cells [12, 13]. The unresponsive cancer patients may need a greater boost of their tumor-specific T cells to achieve more successful immunotherapy with checkpoint inhibitor-blocking antibodies. Based on its dual antiproliferative effect on LLC and DC-maturing effect, we hypothesized that CT could perhaps be a good candidate to induce antitumor immunity in LLC-bearing immunocompetent mice. Indeed, CT together with anti-PD-L1 cured LLC-bearing mice with the induction of subsequent LLC-specific immunity as described in the accompanying paper by Liu et al. [10.1007/s00262-019-02326-8]. However, it remains to be determined [1] whether CT can inhibit the proliferation of other cancer cells such as hepatocellular carcinoma (HCC) cells; [2] whether CT can activate APCs other than DCs, such as macrophages; [3] whether CT can induce tumor-specific immunity in mouse models other than LLC; and [4] to determine the receptor and pathway used by CT to induce adaptive immunity.

In the current study, we investigated the potential antiproliferative effect of CT on Hepa1-6 cells and found that CT inhibited the growth of Hepa1-6 cells by inducing apoptosis through blockade of the JAK2/STAT3 signaling pathway. We also discovered that CT activates macrophages in an M1 polarized direction using the TLR7/MyD88/NF-κB signaling pathway. Furthermore, when treated with a combination of CT and anti-PD-L1, mice with established Hepa1-6 tumors were completely cured, with the generation of Hepa1-6-specific immunity. Thus, CT possesses the dual capacities to inhibit the growth of multiple tumors and promote antitumor immune responses.

## Materials and methods

### Mice and cell lines

C57BL/6, TLR7^−/−^, MyD88^−/−^, and immunodeficient nude mice (8–12 weeks old, female) were kept under specific pathogen-free conditions with water and food given ad libitum.

Hepa1-6 hepatoma cell line (CRL-1830) and EG7 thymoma cell line (CRL-2113) used in the present study were maintained in DMEM (Meditech) supplemented with 10% FBS (Hyclone) and 2 mM l-glutamine, 25 mM HEPES, 100 U/ml penicillin, 100 µg/ml streptomycin, and 50 µM 2-mercaptoethanol at 37 °C in a humidified incubator with 5% CO_2_.

### Cell proliferation assay

Hepa1-6 cells (5 × 10^3^/well) were seeded in triplicate in round-bottomed 96-well plates in complete DMEM (0.2 ml/well) and incubated in the presence or absence of the indicated concentration of CT at 37 °C in a CO_2_ incubator for 48 h. Tritiated thymidine (^3^H-TdR, New England Nuclear, North Billerica, MA) was added at 0.5 μCi/well for the last 4 h of culture. The cultures were harvested on a membrane using a 96-well automatic harvester (INOTECHAG IH-280, Dottikon, Switzerland). The filter mat and scintillation fluid were placed into a bag, which was sealed and assessed for ^3^H-TdR incorporation (CPM) using an automatic MicroBeta counter (Wallac).

A CCK8 (Sigma-Aldrich, St. Louis, MO, USA) assay was performed to assess cell viability in Hepa1-6 cells treated with different concentrations of cucurbitacin I, according to the manufacturer’s instructions.

### Cell cycle and apoptotic assays by flow cytometry (FACS)

The cycle distribution was analyzed by FACS analysis after staining with propidium iodide (PI) solution. Briefly, Hepa1-6 cells were treated with CT for 48 h and fixed with 75% ethanol. Next, the cells were incubated with 500 µL of a solution containing 50 µg/mL PI and 0.1% Triton X-100 in the dark and analyzed by FACS. To further analyze the apoptosis induction effects of CT, the cell apoptosis was detected as described previously [8]. After treatment with CT for 48 h as described above, both attached cells and floating cells were harvested, stained with PI and Annexin V-FITC Apoptosis Detection Kit according to the manufacturer’s instructions, and analyzed by FACS.

### Generation and treatment of bone marrow-derived macrophages (BMM)

Mouse BMM were generated as described previously [14]. To measure surface marker of mouse BMM, adherent cells were grown to confluence in 24-well plates (at about 5 × 10^5^/well) in a CO_2_ incubator in the presence or absence of various reagents at concentrations specified for 48 h before immunostaining.

### Immunostaining and FACS

Cultured BMMs were detached as described by Han et al. [14]. The BMMs were resuspended at 1.0 × 10^6^ cells/1 ml in PBS and then incubated with FITC-anti-mouse CD86 (clone GL1, TONBO Biosciences, San Diego, CA), PE-anti-mouse CD80 (clone 16-10A1, TONBO), Alexa Fluor^®^ 647-anti-mouse CD206 (clone MR5D3, BD Pharmingen). For immunoprofiling of tumor-bearing mice, single cell suspensions of Hepa1-6 tumors or the draining lymph nodes (dLN) at 1 × 10^6^ cells/sample were immunostained with a combination of some of the following antibodies, such as FITC-anti-mouse CD4 (clone GK1.5, Tonbo), PE-anti-mouse CD11b (clone M1/70, BD), PerCP-Cy5-anti-mouse-B220 (clone RA3-6B2, Tonbo), APC-anti-mouse-CD11c (clone HL3, BD), eFluor450-anti-mouse CD45 (clone 30-F11, eBioscience), APC-Cy7-anti-mouse-CD8 (clone 53-6.7, Tonbo), eFluor450-anti-mouse CD44 (clone IM7, eBioscience), APC-anti-mouse CD62L (clone MEL-14, eBioscience), eFluor450-anti-mouse CD8 (clone 53-6.7, eBioscience), and eFluor660-anti-mouse CD107a (clone 1D4B, eBioscience). Data of the stained samples were acquired using an LSR II flow cytometer (BD) and analyzed using the software FlowJo (Tree Star Inc., Ashland, OR).

### Total RNA isolation and cDNA synthesis

Total RNA from mouse BMM was isolated according to the protocol of RNeasy Micro Kit (Qiagen, Hilden, Germany, Cat: 74,004). Total RNA from Hepat1-6 tumors was extracted using TRIzol (Invitrogen, Cat: 1,559,026) and purified using the RNeasy Micro Kit RNA. The purity and concentration of isolated RNA samples were determined by measuring absorption at 260 nm wavelength using an NanoDrop ND-1000 spectrometer (Nanodrop Technologies, Wilmington, DE). cDNA was then synthesized from the RNA using the RT^2^ First Strand Kit (Qiagen, Cat: 330,401).

### Quantitative real-time polymerase chain reaction (qPCR)

qPCR was performed using a LightCycler 480 II (Roche Life Sciences, Branford, CT, USA), the RT^2^ SYBR Green/ROX qPCR Master Mix (Qiagen, Cat: 330,523), and the specific primer pairs (sTable 1). The cycling conditions for the qPCR amplification were: hot start for 10 min at 95 °C; amplification for 40 cycles at 95 °C for 15 s, 55 °C for 35 s, and 72 °C for 30 s. The transcript levels were then normalized to that of a house-keeping gene (i.e., β actin or GAPDH) and then the data were analyzed using the ^ΔΔ^CT method through Qiagen’s GeneGlobe Data Analysis Center.

### Western blot analysis

Western blotting analysis was performed as described previously [15]. Briefly, cells treated with CT for 48 h were lysed in RIPA buffer (Beyotime, Beijing, China). The protein concentrations were quantitated with Pierce™ BCA Protein Assay Kit (Thermo Fisher Scientific, Frederick, MD, USA). Total protein 30 µg/lane were loaded onto SDS–polyacrylamide gels and transferred to polyvinylidene fluoride (PVDF) membranes (Amersham Bioscience, Piscataway, NJ). The membranes were blocked and incubated with (1:1000) rabbit anti-p-STAT3 (Cell Signaling, Cat: 9145L, Tyr 705), anti-STAT3 (Cell Signaling, Cat: 4904S), anti-p-JAK2 (Cell Signaling, Cat: 8082), anti-JAK2 (Cell Signaling, Cat: 3230), anti-I-kBα (Cell Signaling, Cat: 9242), anti-GAPDH (Cell Signaling, Cat: 2118) overnight at 4 °C and (1:2000) horseradish peroxidase-conjugated secondary antibody (Cell Signaling, Cat: 70,741) for 1 h at room temperature. The protein bands were visualized using the G-BOX Chemi system (Syngene, Frederick, MD).

### Cytokine quantitation

TNFα and IL-12p70 in the culture supernatants were quantitated by human and mouse Customary Cytokine Arrays following the manufacturer’s protocol (MesoScale Diagonostics, Rockville, MD).

### Mouse tumor model and treatment

Female mice (C57BL/6, *n* = 5–10, 8–12-weeks old) were injected subcutaneously with 0.1 ml PBS containing Hepa1-6 (2 × 10^7^/ml) or EG7 (2 × 10^5^/ml) or into left or right flank regions as previously reported [16, 17]. The appearance and size of tumors, as well as the mouse body weight, were monitored twice weekly. The length (*L*) and width (*W*) of tumors were measured with a caliper. Tumor size was calculated by the formula: (*L* × *W*^2^)/2. Hepa1-6-bearing mice were treated with intratumoral (i.t.) injection of CT alone or in combination with anti-PD-L1 as indicated. In some experiments, tumor-bearing mice were simultaneously treated with intraperitoneal (i.p.) administration of 200 μg of either control rat IgG (clone 2A3, BioXcell, West Lebanon, NH), anti-mouse CD4 (clone GK1.5, BioXcell), anti-mouse CD8α (clone 53-6.72), or anti-mouse NK1.1 (clone PK136, BioXcell). For the analysis of leukocyte infiltration in Hepa1-6 tumor tissue, the tumors were removed and dissociated into single cell suspensions using an enzymatic cocktail consisting of collagenase I, II, and VI, deoxyribonuclease I, and elastase as previously reported [18].

### Statistical analysis

Student’s *t* tests were performed for parametric comparisons between two groups. A two-way analysis of variance (ANOVA) was used to analyze tumor volume difference between groups. Differences in survival curves were considered statistically significant by the log-rank survival analysis. All experiments were performed at least three times, and the results of one representative experiment or the mean of multiple experiments are shown. All statistical analyses were conducted using GraphPad Prism software (version 7, GraphPad Software, San Diego, CA).

## Results

### Antiproliferative effects of CT on Hepa1-6 hepatoma cells

**Fig. 1:**
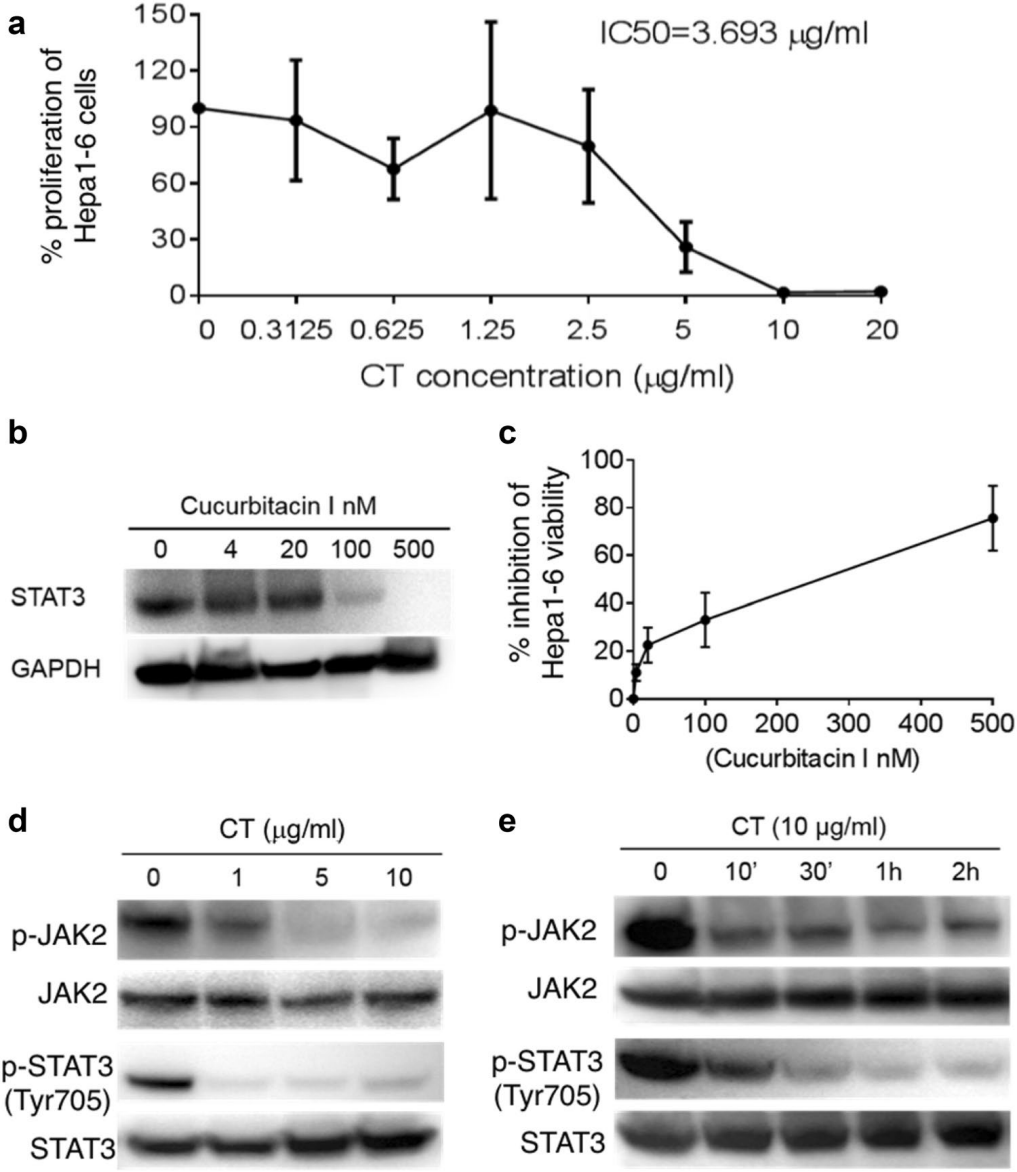
CT inhibition of Hepa1-6 hepatoma cell proliferation. **a** Hepa1-6 cell proliferation was measured using ^3^H-TdR incorporation and presented as the average (mean ± SD) of ^3^H-TdR incorporation of triplicate wells. The % proliferation was calculated as (CPM with compound—CPM blank) × (CPM without compound—CPM blank) × 100. **b** Hepa1-6 cells treated with different concentrations of cucurbitacin I for 48 h were solubilized in 1 × SDS sample buffer at 10^7^/ml, separated on a gradient gel, and analyzed for STAT3 by Western blot. The same membrane was stripped and re-probed with anti-GAPDH. **c** A CCK-8 assay was performed to assess cell viability in Hepa1-6 cells treated with different concentrations of cucurbitacin I for 48 h. All data are averages (mean ± SD). **d**, **e** Hepa1-6 cells grown in 6-well plates were treated with CT at various concentrations for 2 h (**d**) or at specified concentration and time (**e**). The samples were analyzed by Western blot for phospho-JAK2 or phospho-STAT3. The same membranes were stripped and re-probed with anti-JAK2 or anti-STAT3, respectively

To assess the antiproliferative effect of CT on the Hepa1-6 cells, twofold increases in concentrations from 0.3125 to 20 µg/ml (ranging from 1.05 to 67.2 µM) of CT were incubated for 48 h with cultured Hepa1-6 cells. As shown in Fig. 1a, treatment with CT markedly inhibited the in vitro proliferation of Hepa1-6 cells in a concentration-dependent manner above 0.3125 µg/ml (1.05 µM). In comparison, Hepa1-6 cells treated with PBS containing 1% DMSO as a control did not inhibit ^3^H-TdR incorporation. Incubation of Hepa1-6 cells with 2.5 or 5 µg/ml (8.4 and 16.8 µM) CT for 48 h reduced the percentages of surviving cells relative to controls to 74.8 ± 12.6 and 30.4 ± 8.3%, respectively, indicating that CT potently inhibited the proliferation of the cells at low molar concentrations. The IC50 value of CT was approximately 3.693 µg/ml (12.5 µM) on Hepa1-6 cells.

Treatment of Hepa1-6 cells with CT at 1 or 5 µg/ml (3.36 and 16.8 µM, respectively) did not cause cell-cycle arrest (sFig. 1). It has been reported that the antiproliferative effect of CT on melanoma cells is due to induction of apoptosis [7]. The percentage of apoptotic cells following incubation of Hepa1-6 cells with CT for 48 h was therefore determined by FACS. As shown in sFig. 2, the apoptotic cell fractions were increased following treatment with CT in comparison with the control group. The fraction of apoptotic cell in the control group was only 0.24%. The addition of 1 and 5 µg CT to the Hepa1-6 cells increased the apoptotic cell fractions to 5.25 ± 1.2 fold (*p *< 0.01) and 14.79 ± 2.8 fold (*p *< 0.01) higher, respectively, than those of the control group. Therefore, CT inhibited Hepa1-6 proliferation perhaps by induction of apoptosis.

STAT3 signaling plays a pivotal role in tumor growth [19–21]. Figure 1b shows that Hepa1-6 cells constitutively express STAT3, which can be inhibited by Cucurbitacin I (STAT3 inhibitor) in a dose-dependent manner. In addition, Cucurbitacin I also dose-dependently inhibited the viability of Hepa1-6 cells (Fig. 1c). These data indicate that inhibition of STAT3 has an antiproliferative effect on Hepa1-6 cells. We therefore investigated whether CT also inhibits the STAT3 signaling pathway in Hepa1-6 cells. Our results in Fig. 1d indicated that after incubation for 2 h with increasing concentrations of CT, the phosphorylation of STAT3 at Tyr705 was inhibited beginning at the lowest concentrations (1 μg/ml, 3.36 µM of CT) without marked effects on the total protein level of STAT3. Following exposure to 10 µg/ml of CT for 2 h, p-STAT3 was almost undetectable in the Hepa1-6 cells. The effect of CT on inhibition of JAK2, the upstream kinases of STAT3, was next investigated. The results indicated that phosphorylation level of JAK2 protein was also decreased by CT at the same concentrations (Fig. 1d). Furthermore, CT inhibited STAT3 and JAK2 phosphorylation within 10 min in the Hepa1-6 cells (Fig. 1e). Thus, CT inhibited Hepa1-6 cells proliferation by suppressing the JAK2/STAT3 signaling pathway.

### CT promotes M1 polarization of mouse BMM in vitro

**Fig. 2:**
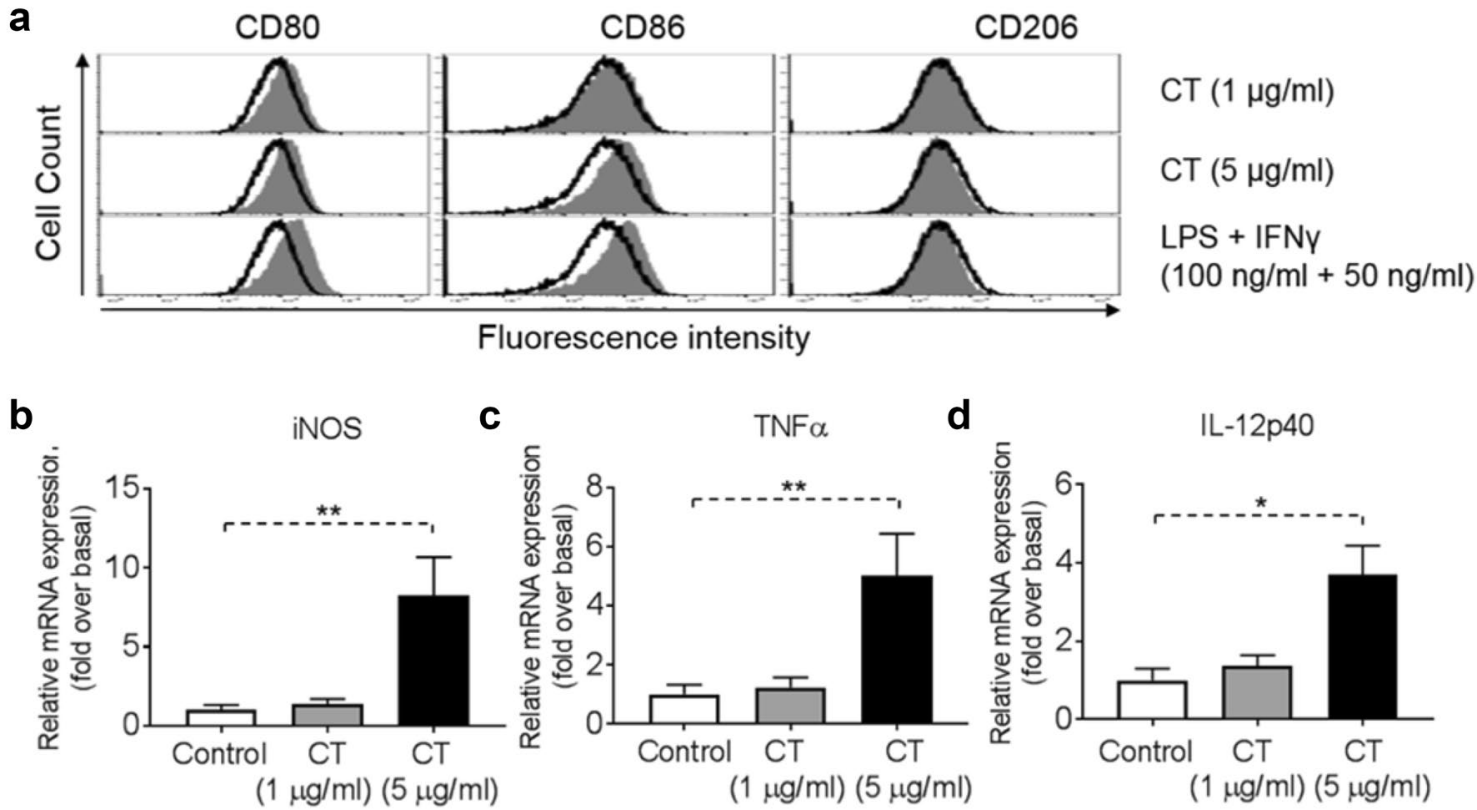
Activation of bone marrow-derived macrophage (BMM) by CT. **a** Mouse BMM were incubated in the absence (sham-treated) or presence of CT at 1 or 5 μg/ml (CT-treated) or 100 ng/ml LPS + 50 ng/ml IFNγ (positive control) for 48 h before immunostaining and flow cytometric analysis. The position of quadrants was determined by BMM stained with FITC- and PE-conjugated isotype-matched control antibodies. The density plots of the results of one experiment representative of three are shown (Black line = un-treated, Grey area = treated). **b**–**d** BMM (5 × 10^5^ cells/ml) were incubated in triplicate in the absence or presence of CT at the specified concentrations for 48 h before the supernatant was harvested for the measurement of iNOS (**b**), TNFα (**c**) and IL-12p40 (**d**) mRNA levels by qPCR. The average (mean ± SD) of two experiments using independent donors is shown. ^*^p < 0.05 and ^**^p < 0.001

We performed a thorough analysis of the phenotype of mouse BMM upon treatment with various concentrations of CT (1 or 5 µg/ml). As shown by Fig. 2a, stimulation of mouse BMM by 1 µg/ml of CT had little effect, but 5 µg/ml of CT induced CD80, CD86, but not CD206, markers, typical of M1 polarization. Additionally, treatment of BMM with 5 µg/ml CT for 48 h increased the mRNA expression levels of iNOS (Fig. 2b), TNFα (Fig. 2c) and IL-12p40 (Fig. 2d) as compared with control. Altogether, these results indicated that CT activated BMM in the direction of M1 polarization.

**Fig. 3:**
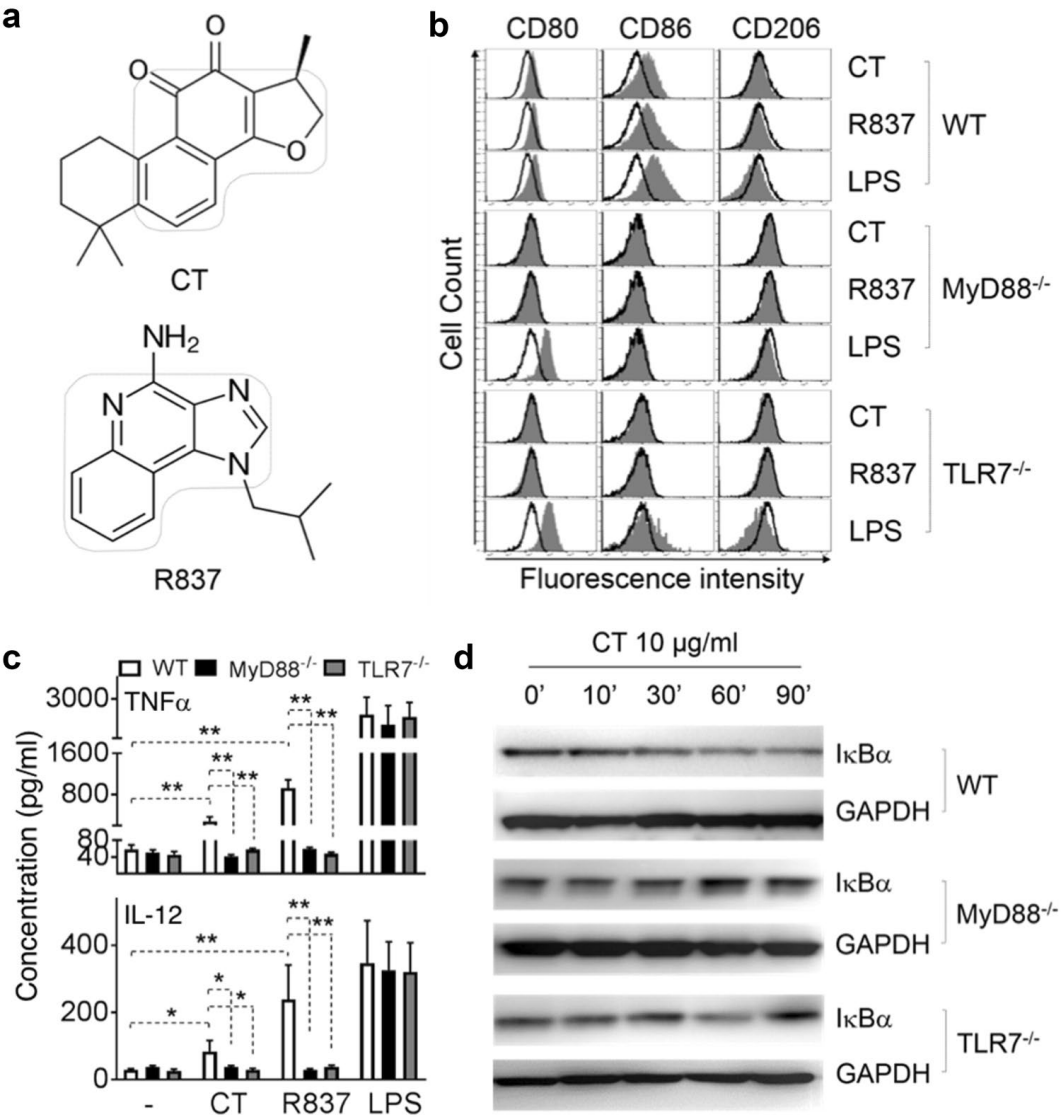
Effect of CT on wild-type (WT), MyD88^−/−^, and TLR7^−/−^ mouse BMM. **a** Structure of CT and R837. **b** WT (C57BL/6), MyD88^−/−^, and TLR7^−/−^ mouse BMM were incubated in a CO_2_ incubator for 48 h with CT, R837 or LPS at specified concentrations before they were analyzed for the expression of CD80, CD86, and CD206 by flow cytometry. Shown are the overlay histograms of sham (black line) and treated (shadow) BMM. **c** Mouse BMM were cultured in the absence (sham) or presence of 5 μg/ml CT for 48 h before the supernatants were harvested for the measurement of indicated cytokines. Shown is the average (mean ± SD) of three independent experiments. ^*^p < 0.05 and ^**^p < 0.001. **d** WT, MyD88^−/−^, and TLR7^−/−^ mouse BMM under M1 macrophage polarization condition (100 ng/ml LPS + 50 ng/ml IFNγ) for 24 h, followed by 10 μg/ml CT treatment for indicated time periods. The samples were analyzed by Western blot for I-κBα. The same membrane was re-probed with anti-GAPDH after stripping

CT induction of DC maturation depends on MyD88 [10.1007/s00262-019-02326-8], suggesting that CT may use a receptor belonging to either Toll-like receptor or IL-1 receptor superfamily. Comparison of structures of CT and R837/imiquimod reveals that they have similar backbones (Fig. 3a), suggesting that CT might use the same receptor that R837 uses. We therefore investigated whether CT required MyD88 and TLR7 for CT to induce M1 polarization of BMM. To this end, WT, MyD88^−/−^, and TLR7^−/−^ BMM were treated in parallel with CT at 5 μg/ml, R837 (100 ng/ml) or LPS (100 ng/ml) as positive controls for 48 h. The capacity of CT to induce expression of CD80 and CD86 was completely deficient in MyD88^−/−^ BMM and TLR7^−/−^ BMM (Fig. 3b). Furthermore, the production of TNFα and IL-12p70 was markedly reduced in MyD88^−/−^ and TLR7^−/−^ BMM as compared with WT (Fig. 3c). These results demonstrated that CT induced mouse BMM polarization toward M1 phenotype in a MyD88 and TLR7-dependent manner.

To further identify the consequent downstream signaling events induced by CT, the effect of CT at 10 μg/ml on the activation of NF-κB in BMM was determined (Fig. 3d). CT decreased the levels of I-κBα protein within 30 min (Fig. 3d, upper panel). Since degradation of I-κBα frees the p50/p65 dimer of NF-κB and enables its nuclear translocation, the data indicate that CT promoted NF-κB activation.

### Therapeutic antitumor effect of CT on Hepa1-6 tumors

**Fig. 4:**
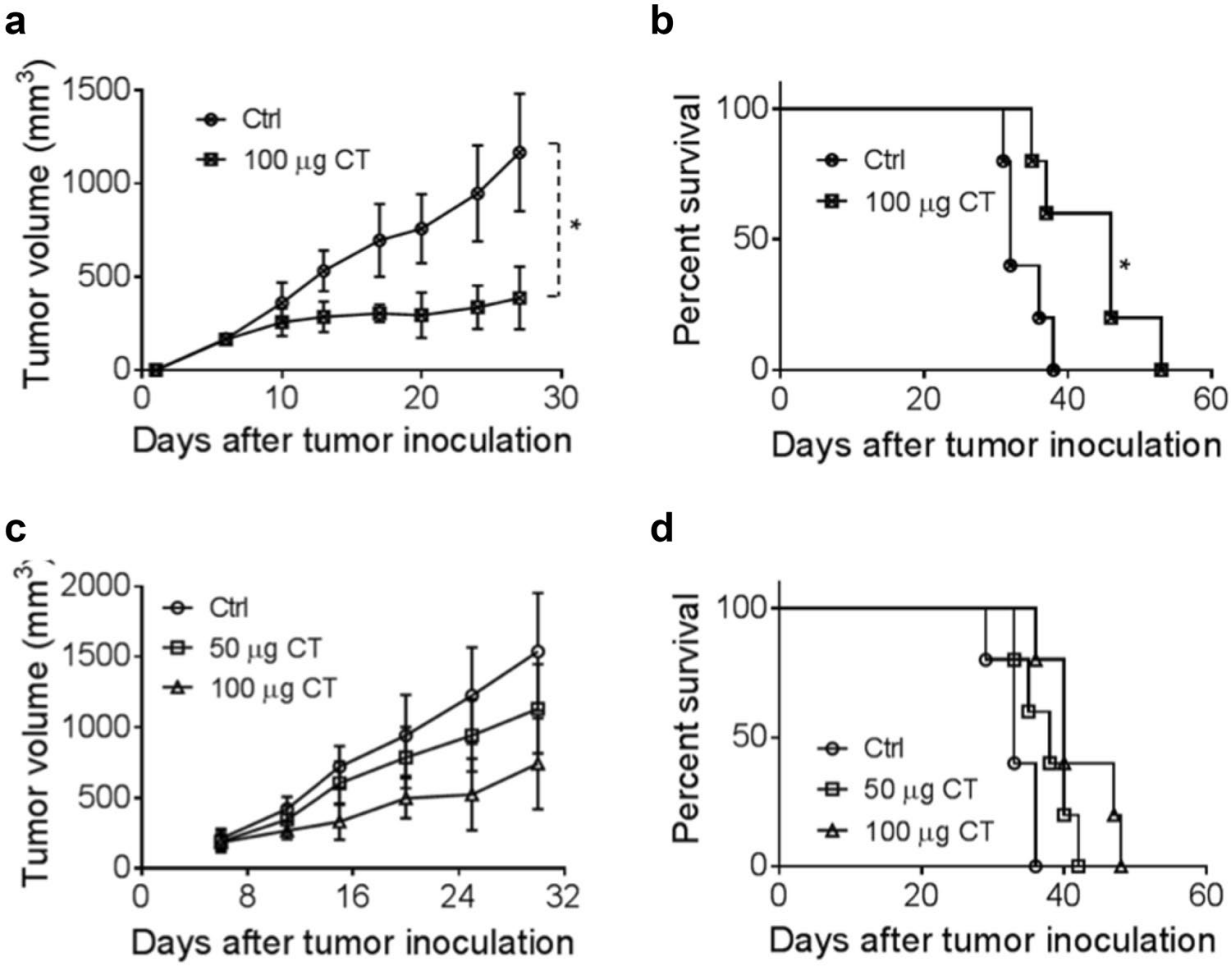
The in vivo therapeutic effect of CT on smaller Hepa1-6 tumors. **a**, **b** C57BL/6 mice (female, 8 weeks old, *n* = 5) were inoculated s.c. with 2 × 10^6^/mouse of Hepa1-6 cells in the right flank on day 1 and the formation of tumors was monitored. When tumors reached approximately 0.5 cm in diameter (day 5 ~ 6), tumor-bearing mice were treated with PBS and CT 100 μg/injection/tumor twice every other day for 2 weeks. Tumor growth (mean ± SD) and survival were monitored and plotted. The results of one experiment representative of three are shown. ^*^p < 0.05 and ^**^p < 0.001. **c**, **d** immunodeficient nude mice (female, 8 weeks old, *n* = 5) were subjected to the same procedures as **a** and **b**

To investigate the contribution of the immune response to the antitumor effect of CT, we compared immunocompetent versus immunodeficient mice. CT was injected intratumorally (i.t.) into C57BL/6 mice (Fig. 4a, b) and nude mice (Fig. 4c, d) bearing Hepa1-6 tumors of approximately 0.5 cm in diameter. The i.t. route of administration, dose, and dosing schedule of CT was based on previous reports [18, 22, 23]. CT at 100 µg/mouse administered i.t. biweekly for 2 weeks markedly suppressed the growth of established hepa1-6 tumors in C57BL/6 mice (Fig. 4a) (*p *< 0.05). Moreover, the overall survival rate of CT-treated group was markedly higher than that of the control group of C57BL/6 mice (Fig. 4b). A number of previous reports have shown that CT can inhibit the growth of human xenograft tumor in nude mice [6, 24]. Indeed, CT also dose-dependently slowed down the growth of Hepa1-6 tumors in nude mice and moderately promoted the survival of Hepa1-6-bearing nude mice (Fig. 4c, d). These results indicate that CT is more effective for tumor suppression in immunocompetent mice than in immunodeficient mice, attesting to the importance of the immune-activating activity of CT in its antitumor effect. Nevertheless, the cytotoxic effect of CT may serve to augment the supply of tumor antigens available to the immune system and presentation for phagocytic uptake by APCs including dendritic cells and macrophages.

**Fig. 5:**
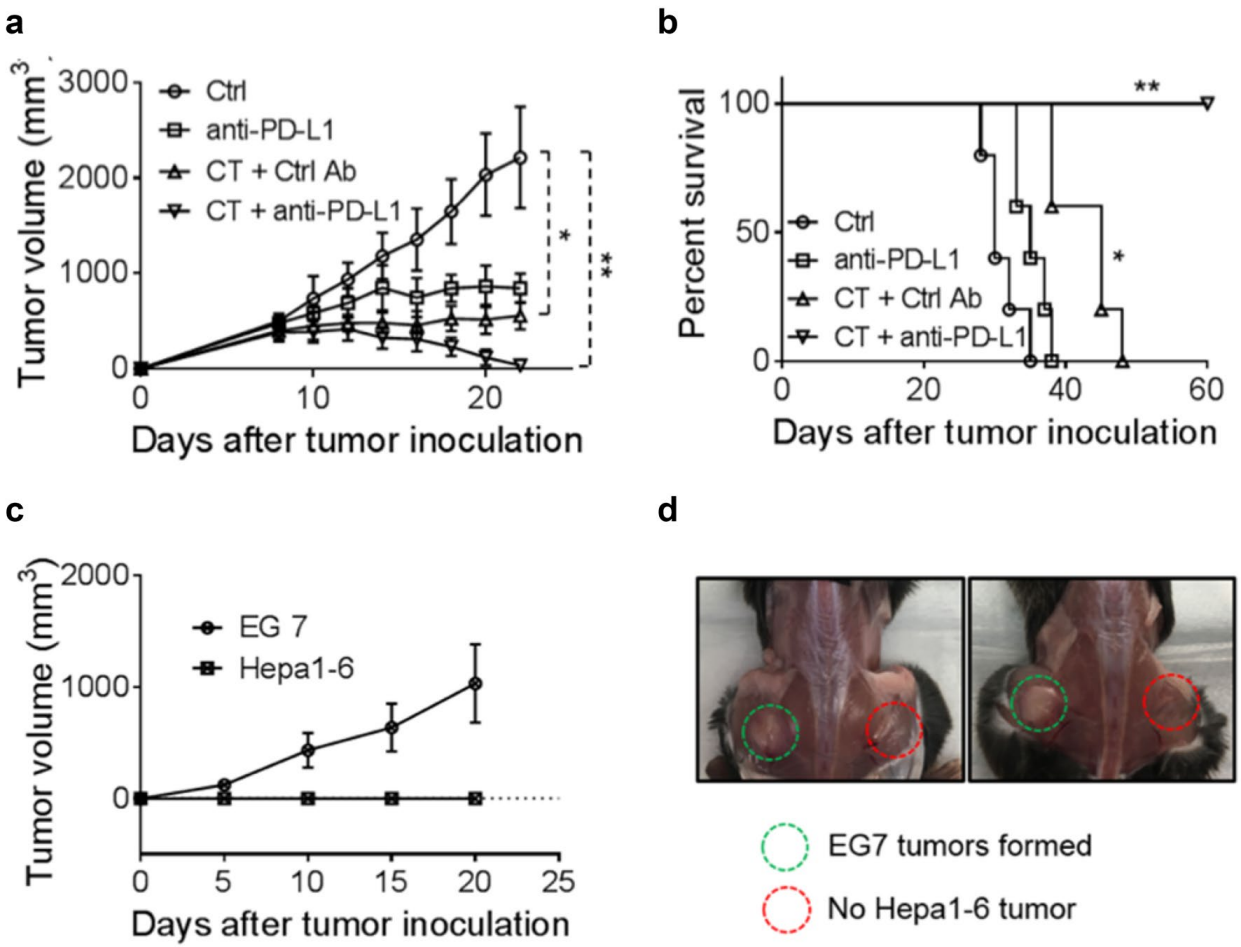
The effect of anti-PD-L1 together with CT on mice harboring large Hepa1-6 tumors. **a**, **b** C57BL/6 mice (female, 8 weeks-old, *n* = 5) were inoculated s.c. with 2 × 10^6^/mouse of Hepa1-6 cells in the right flank on day 1 and the formation of tumors was monitored. When tumors reached approximately 1.0 cm in diameter (day 9 ~ 10), tumor-bearing mice were treated with i.t. injection of CT (100 μg/mouse) every other day or CT combined with i.t. injection of anti-PD-L1 antibody (10 μg/mouse) twice weekly for 2 weeks. Tumor growth (mean ± SD) and survival were monitored and plotted. Shown are the results of one experiment representative of three. ^*^p < 0.05 and ^**^p < 0.001. **c**, **d** The mice cured of hepatoma by the treatment with CT and anti-PD-L1 were inoculated s.c. with 2 × 10^6^/mouse of Hepa1-6 cells in the right flank and 2 × 10^5^/mouse of EG7 cells in the left flank. The growth of tumors on both flanks was monitored and graphed

The failure of CT by itself to halt the growth of Hepa1-6 tumors might be due to the immunosuppressive tumor microenvironment that hindered the elimination of tumor cells. Therefore, anti-PD-L1 was added to the therapeutic regimen along with CT to treat C57BL/6 mice bearing Hepa1-6 tumors of approximately 1.0 cm in diameter. As shown in Fig. 5a, CT (100 μg/mouse) and anti-PD-L1 (10 μg/mouse) treatment by themselves only delayed the growth of Hepa1-6 tumors in comparison with controls. The combination of eight i.t. doses of CT and four i.t. doses of anti-PD-L1 completely inhibited the growth of Hepa1-6 tumors of approximately 1.0 cm in diameter. Hepa1-6-bearing mice treated with this combination became long-term tumor-free survivors (Fig. 5b). Mice, which were tumor-free for 2 months, were inoculated s.c. with Hepa1-6 cells into the right flank and EG7 (a C57BL/6 lymphoma cell line) into the contralateral flank. All mice formed EG7 tumors, whereas the mice did not develop any Hepa1-6 tumor (Fig. 5c, d). Thus, mice cured of the initial large Hepa1-6 tumors became specifically resistant to challenge with Hepa1-6, but not to an unrelated EG7 tumor, indicative of the development of long-term systemic specific antitumor immunity.

### The therapeutic treatment Hepa1-6 tumors by CT was accompanied by induction of antitumor immune responses dependent on T lymphocytes

**Fig. 6:**
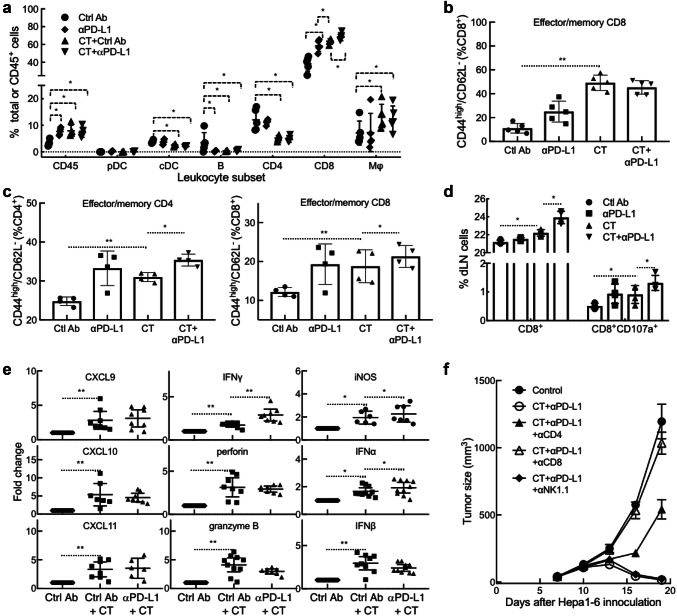
Immunological profiling of the therapeutic effect of CT on Hepa1-6-bearing mice. **a**–**e** Hepa1-6-bearing C57BL/6 mice (*n* = 5) were treated as described in Fig. 5. Twenty-four hours after the third treatment, the tumors and draining lymph nodes (dLNs) were removed and subsequently dissociated into single cell suspension. **a** Tumor cell suspensions (1x10^6^/sample) were immunostained and analyzed by flow cytometry as detailed in sFig. 3. Shown is the CD45^+^ leukocyte percentage in total cell suspension or specific leukocyte percentage in CD45^+^ leukocytes. *pDC* plasmacytoid DC, *cDC* conventional DC, *Mϕ* macrophage. **b** Effector/memory CD8 (CD44^high^/CD62L^−^) cells in the tumors analyzed by flow cytometry as detailed in sFig. 4. **c** Effector/memory (CD44^high^/CD62L^−^) CD4 and CD8 T cells in the tumors analyzed by flow cytometry as detailed in sFig. 5. **d** dLN cells cocultured with Hepa1-6 monolayers were immunostained and analyzed by flow cytometry as detailed in sFig. 6. Shown is the percentage of CD8^+^ or CD8^+^/CD107a^+^ cells. **e** qPCR measurement of the target genes of Hepa1-6 tumors (*n* = 7 ~ 10) resected 24 h after the third treatment (fold change). **f** Hepa1-6-bearing mice were treated with i.t. injection of CT (100 mg/mouse) plus anti-PD-L1 (αPD-L1, 10 mg/mouse) without or with i.p. administration of depleting antibody against mouse CD4, CD8, or NK cells. Tumor growth (mean ± SD) was monitored and plotted. ^*^p < 0.01 and ^**^p < 0.001

To increase our understanding on how CT promotes antitumor immunity, Hepa1-6 tumors and draining lymph nodes (dLN) of tumor-bearing mice were analyzed for leukocyte infiltration, memory T cells, and functional CTLs. Flow cytometry analysis of the single cell suspensions of treated tumors revealed that CT treatment dramatically enhanced the infiltration of CD45^+^ leukocyte, CD8 T cells, and macrophages (defined as CD11b^+^/CD11c^−^/CD45^+^) into the tumor tissue (Fig. 6a and sFig. 3). In contrast, the frequency of conventional DC (CD11c^+^/B220^−^/CD45^+^), B cells (B220^+^/CD45^+^), and CD4 T cells in Hepa1-6 tumors was reduced (Fig. 6a and sFig. 3). The reduction of cDC content in response to CT treatment is also seen in another mouse tumor model [10.1007/s00262-019-02326-8], which might be due to induction of DC maturation since matured DCs migrate to dLNs as previously reported [18]. Combined treatment with CT plus anti-PD-L1 further increased the infiltration of CD8 T cells in Hepa1-6 tissue, suggesting the added immunotherapeutic efficacy of anti-PD-L1 on Hepa1-6 tumors (Fig. 6a). When the tumor-infiltrating CD8 T cells were further analyzed, the abundance of effector/memory CD8 (CD44^high^/CD62L^−^) was significantly elevated (Fig. 6b and sFig. 4). CT treatment also resulted in an increase in the frequency of effector/memory CD4 and CD8 T cells in Hepa1-6 dLNs, which was further elevated by combined treatment with anti-PD-L1 (Fig. 6c and sFig. 5). To determine if functional anti-Hepa1-6 CTLs were induced by CT treatment, dLN cells were cocultured with Hepa1-6 cells ex vivo for 18 h and the resultant CD8 cells were analyzed for the expression of CD107a. As shown by Fig. 6d and sFig. 6, the frequencies of both CD8^+^ and CD8^+^/CD107a^+^ in the dLNs were significantly increased by CT treatment, which was further elevated by combined treatment with anti-PD-L1. Therefore, CT treatment of Hepa1-6-bering mice promoted generation of memory and Hepa1-6-specfic functional CD8 T cells in the dLNs and infiltration of CD8 T cells in the tumors, a profile characteristic of antitumor immunity.

Measurement of the expression of a panel of genes in Hepa1-6 tumors showed that CT treatment significantly upregulated the expression of CXCL9, CXCL10, CXCL11, IFNγ, perforin, granzyme B, iNOS, IFNα, IFNβ, IL-12p40, and TNFα in the tumor tissue (Fig. 6e and sFig. 7). CXCL9-11, chemokines critical for the recruitment of Th1 T cells and CTLs to the peripheral tissue, were likely responsible for attracting CD8 T cells to Hepa1-6 tumors. Elevated levels of IFNγ, perforin, and granzyme B was in agreement with elevated frequencies of functional CD8 CTLs in Hepa1-6 tumors (Figs. 6ab and sFig. 3). Elevated expression of iNOS, IFNα, IFNβ (Fig. 6e), IL-12p40, and TNFα (sFig. 7) was indicative of M1 activation of macrophages and/or DCs in Hepa-6 tumors of CT-treated mice. CT treatment did not significantly promote the expression of IL-4, IL-13, IL-10, and TGFβ1 in Hepa1-6 tumors (sFig. 7). The expression of IL-17a in Hepa1-6 tumor tissue was significantly downregulated by CT treatment (sFig. 7). Overall, the pattern of gene expression indicates that CT treatment resulted in a tumor microenvironment indicative of macrophage differentiation towards M1, DC activation, Th1 polarization, and CTL infiltration.

To further determine whether the therapeutic antitumor effect of CT was dependent on the generation of antitumor immunity in this tumor model, mice bearing Hepa1-6 tumors were treated with CT plus anti-PD-L1 without or with depleting antibody against mouse CD4, CD8, or NK cells. As shown by Fig. 6f, the curative antitumor effect of CT plus anti-PD-L1 was completely abolished by depleting CD8 T cells, while depleting CD4 T cells partially inhibited the immunotherapeutic effect. Depleting NK cells did not inhibit the immunotherapeutic effect of CT plus anti-PD-L1 on Hepa1-6 (Fig. 6f), further indicating that CT regression of Hepa1-6 tumors by induction of adaptive immunity.

## Discussion

In this study, we developed a successful immunotherapeutic vaccination regimen of Hepa1-6 tumors based on cytotoxic effect and the immune-activating effect of CT with potentiation of the antitumor therapeutic effects by addition of the checkpoint inhibitor (anti-PD-L1).

In vitro and in vivo studies have demonstrated that CT inhibits cell proliferation in a variety of cancer cell lines [6, 10, 25–29]. More recent reports have shown that CT inhibits the proliferation of lung cancer cells and leukemia cells by affecting insulin growth factor-1 receptor signaling and protein synthesis, respectively [30, 31]. In our study, CT inhibited the growth of Hepa1-6 both in vitro and in vivo (Figs. 1, 4 and 5). It has been reported that CT induced a G_1_/G_0_ cell-cycle arrest in rhabdomyosarcoma (Rh30) and prostate cancer (DU145) cells by downregulating expression of cyclin D1 and phosphorylation of retinoblastoma protein (Rb) [25], but that CT induced a G_2_/M cell-cycle arrest in lung (A549) cells via upregulating expression of cyclin-dependent kinases (CDK) [6]. The present study showed that CT did not affect the cell cycle of Hepa1-6 cells, suggesting that CT inhibited Hepa1-6 cells’ proliferation by another pathway.

CT has been reported to induce cell death in tumor cells [6]. In the previous study we showed that CT was antiproliferative for mouse LLC cells by upregulating p53 and downregulating cyclin B1 and Cdc2 and consequently inducing G2/M cell-cycle arrest [10.1007/s00262-019-02326-8]. In the present study, CT inhibited proliferation by the induction of apoptosis in the Hepa1-6 cells (sFig. 2). Previous studies have shown that CT suppresses the proliferation of ovarian cancer cells and induced apoptosis of pancreatic as well as prostate cancer cells via the STAT3 signaling pathway [8, 10, 32–34]. STAT3, regulated by Janus kinases (JAKs), is constitutively activated in most human malignant tumors, and is involved in the proliferation, angiogenesis, immune evasion and has anti-apoptotic effects in cancer cells [35], including HCC [36]. In our study, CT decreased STAT3 phosphorylation in a dose- and time-dependent manner. CT can affect the STAT3 signaling pathway either directly or by alterations of certain upstream regulators [14, 22]. Our study demonstrated that CT also markedly inhibited the activities of JAK2 when the duration of CT exposure was increased to 2 h. Thus, the inhibition of JAK2/STAT3 signaling pathway may provide significant therapeutic benefits to HCC patients. On the other hand, the tumor was only minimally inhibited in nude mice, but more suppressed in immunocompetent mice by CT. This indicates that the antiproliferative effect is less contributory to inhibiting the tumor than the immune effects of CT. Nevertheless, these two effects may cooperate in suppressing tumors in immunocompetent mice.

TAMs are derived from circulating monocyte precursors [37] and are important regulators of tumorigenesis [38]. It has been well documented that TAMs promote the development of tumor, and their infiltration is highly correlated with poor prognosis [39–42]. Furthermore, Fan QM et al. report that TAM represents a dominant myeloid population infiltrating Hepa1-6 tumors [43]. However, macrophages with an M1 phenotype exhibit phagocytic and antigen-presenting activity, produce Th-1-activating cytokines, and mediate cytotoxic functions, including anticancer activity. Based on our observations of greater antitumor responses by immunocompetent than immunodeficient mice to CT in vivo (Fig. 4), we hypothesized that CT, in addition to activating DCs [10.1007/s00262-019-02326-8] might also influence macrophage polarization. Our data show that CT was capable of promoting BMM polarization toward M1 phenotype in vitro with upregulation of CD80 and CD86, and the production of TNFα and IL-12p40 proinflammatory cytokines (Fig. 2). In addition, Hepa1-6 tumors of mice treated with CT elevated the levels of expression of iNOS, IFNα, IFNβ, IL-12p40, and TNFα, but not IL-10 or TGFβ1 (Fig. 6e and sFig. 7), further indications that CT treatment activated macrophages toward M1 type in vivo. Given the APC function of macrophages and the contribution of M1 macrophages in potentiating antitumor immunity, it is presumed that activation of macrophages toward M1 type by CT plays a role in its immunotherapeutic effects on Hepa1-6 tumors.

To determine the signaling pathway utilized by CT, we have identified a receptor for which it acts as an agonist. Using TLR7^−/−^ and MyD88^−/−^ mouse BMM, we found that the absence of TLR7 and MyD88 adaptor molecule blocked induction of M1 polarization and the production of TNFα and IL-12 proinflammatory cytokines by CT (Figs. 3b–d). These findings indicate that signaling in response to CT is TLR7/MyD88-dependent. The activation of MyD88, a key adaptor protein that participates in propagation of TLR downstream signal transduction pathways in turn leads to subsequent activation of NF-κB. Our data show that CT treatment downregulated I-κBα both in mouse BMM and human HEK293 cell expressing TLR8 genes (data not shown), which enables p50/p65 complex to translocate from the cytosol to the nucleus, bind to promoters and activate NF-κB upregulation of the production of proinflammatory cytokines. TLR7^−/−^ and MyD88^−/−^ mouse BMM failed to show downregulation of I-κBα by CT (Fig. 3d). Thus, stimulation of mouse BMM by CT to produce TNF-α and IL-12p40 proinflammatory cytokines required TLR7/MyD88/NF-κB signaling pathway. However, our study does not rule out the possibility that CT may bind and form complexes with some available endogenous ligands for TLR7 and thus induce TLR7 activation.

Immunosuppression is a huge challenge for antitumor therapy. M2-polarized TAMs are well known to directly inhibit the immune response of CD8^+^ T cells via the production of immunosuppressive factors such as IL-10 and TGF-β [44]. Moreover, secreted factors from tumor cells also increase the expression of programmed cell death 1 ligand (PD-L1) in monocytes and macrophages and cytotoxic T lymphocyte antigen 4 (CTLA-4) ligands are constitutively expressed on regulatory T cells [45]. Inhibitory signals from these immune checkpoints suppress the proliferation of CD8^+^ T cells and weaken the tumoricidal activity of cytotoxic T cells. In our study, mice bearing established s.c. Hepa1-6 tumors were cured by a combination of CT and anti-PD-L1 and the resultant tumor-free mice exhibited Hepa1-6-specific antitumor immune responses and immunological memory (Fig. 5). Thus, a reasonable hypothesis would be that CT activates tumor-infiltrating APCs including macrophages and DCs that lead to the activation of T cells, whose adaptive antitumor activity could be further enhanced by M1 polarization of macrophages and reduction of immune suppression through treatment with anti-PD-L1. Indeed, CT treatment of Hepa1-6-bearing mice resulted in the selective upregulation of an array of genes indicative of DC maturation, activation of TAMs toward M1 type, Th1 polarization, and generation of antitumor immune defense, including CXCL9-11, IFNγ, perforin, granzyme B, iNOS, IFNα, IFNβ, IL-12p40, and TNFα in the tumor tissue (Fig. 6e and sFig. 7). Additionally, CT treatment promoted the generation of effector/memory T cells and Hepa1-6-specific functional CTLs in dLNs of Hepa1-6-bearing mice (Fig. 6c, d and sFigs. 5–6). The curative anti-Hepa1-6 therapeutic effect of treatment with CT plus anti-PD-L1 was dramatically reduced by depleting CD8 T or CD4 cells, further substantiating the notion that induction of adaptive antitumor T cell immunity is a major contributor of CT’s antitumor effect.

In conclusion, the present study suggests that CT is a potential therapeutic for the treatment of human hepatoma. However, because of its short half-life, CT has not been used yet in clinical trials as a cancer therapeutic. Despite short half-life, the in vivo administration of CT together with a checkpoint inhibitor successfully eradicated the tumor and induced a long-term immunity in mice. Further investigations on how to improve the stability and efficacy of CT are warranted.

### Electronic supplementary material

Below is the link to the electronic supplementary material.

Supplementary material 1 (PDF 780 kb)

## Abbreviations

APCs: Antigen-presenting cells
BMM: Bone marrow-derived macrophages
CDKs: Cyclin-dependent kinases
CT: Cryptotanshinone
CTLA-4: Cytotoxic T lymphocyte antigen 4
DC: Dendritic cell
HCC: Hepatocellular carcinoma
JAKs: Janus kinases
LLC: Lewis lung carcinoma
PD-L1: Programmed cell death 1 ligand
PI: Propidium iodide
qRT-PCR: Quantitative real-time polymerase chain reaction
RNA: Ribonucleic acid
TAMs: Tumor-associated macrophages
TCM: Traditional Chinese medicine
^3^H-TdR: Tritiated thymidine
