# Supplementary material for: Inhibition of murine hepatoma tumor growth by cryptotanshinone involves TLR7-dependent activation of macrophages and induction of adaptive antitumor immune defenses
Source: Cancer Immunol Immunother. 2019 Jun 3;68(7):1073–85. doi: 10.1007/s00262-019-02338-4 (PMC6584221; doi:10.1007/s00262-019-02338-4)
Supplement: Supplementary file 1 — Supplementary material 1 (PDF 780 kb) [file 262_2019_2338_MOESM1_ESM.pdf]

Supplemental figures and table

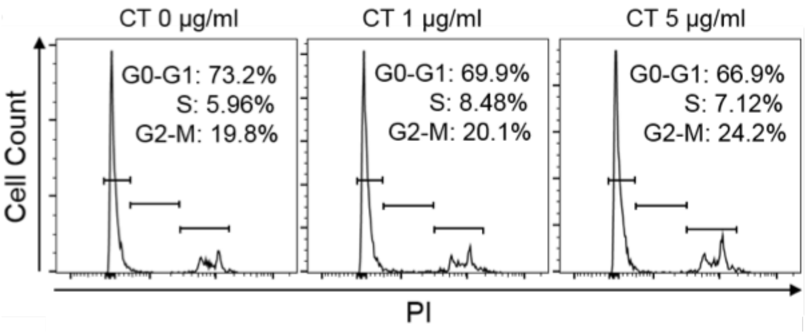

sFig. 1. Cell cycle analysis of CT-treated Hepa-6 cells. Synchronized Hepa1-6 cells incubated with CT for 48 h in a CO_2 incubator were stained with PI and subjected to cell cycle analysis by flow cytometry.

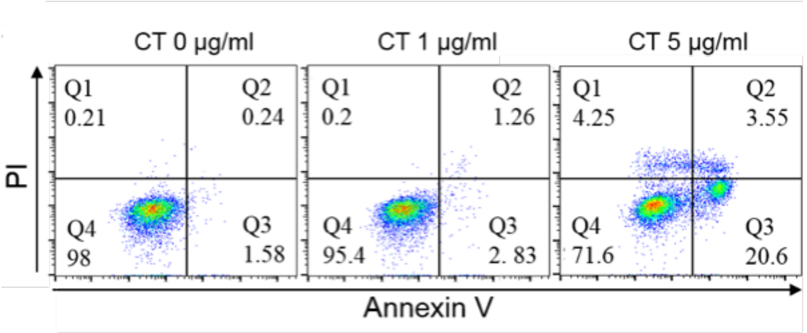

sFig. 2. Determination of cell death of CT-treated Hepa1-6 cells. Hepa1-6 cells seeded in a 12-well plate at 3×10^5/ml/well were incubated with indicated concentrations of CT for 48 h in a CO_2 incubator and before they were harvested, and stained with Annexin V and PI, and analyzed by flow cytometry.

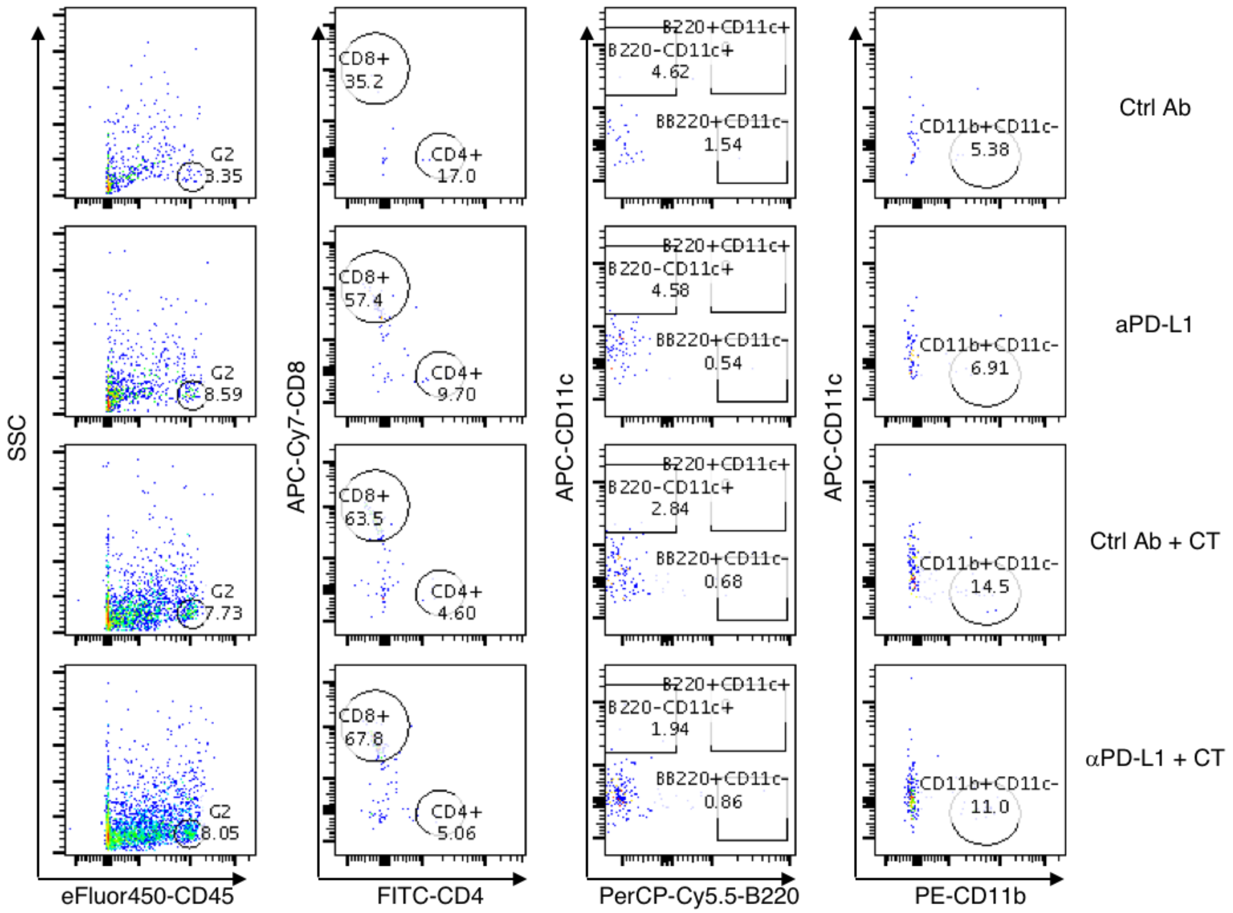

sFig. 3. Flow cytometry determination of leukocyte subsets in the tumors of treated Hepa1-6-bearing mice. Tumors removed from Hepa1-6-bearing C57BL/6 mice 24 h after the 3rd treatment with CT (100 μg/mouse) and anti-PD-L1 (αPD-L1, 10 μg/mouse) alone or in combination were enzymatically dissociated into single cell suspension. The single cell suspensions (1x10^{6}/sample) were immunostained with labeled antibodies against mouse CD45, CD4, CD8, CD11b, CD11c, and B220. CD4 T cells, CD8 cells, B cells (B220^{+}/CD11c^{-}), cDCs (CD11c^{+}/B220^{-}), pDCs (CD11c^{+}/B220^{+}) and macrophage (Mϕ, CD11b^{+}/CD11c^{-}) were analyzed by gating on CD45^{+} populations. Shown are plots of one mouse from each treatment group.

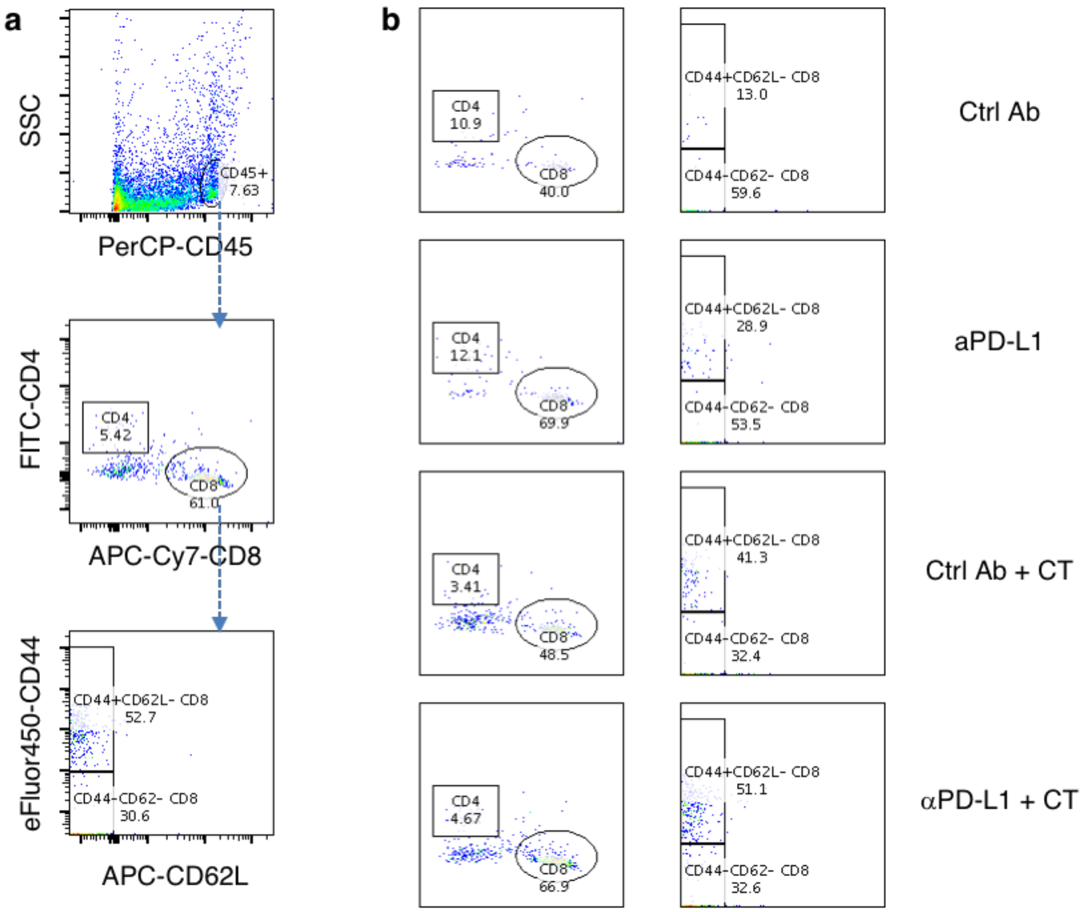

sFig. 4. Flow cytometry analysis of effector/memory ($CD62L^{-}/CD44^{high}$) CD8 T cells in the tumors of treated Hepa1-6 Hepa1-6-bearing mice. removed from Hepa1-6-bearing C57BL/6 mice 24 h after the 3rd treatment with CT (100 μg/mouse) and anti-PD-L1 (αPD-L1, 10 μg/mouse) alone or in combination were enzymatically dissociated into single cell suspension. The single cell suspensions ($1x10^{6}$/sample) were immunostained with labeled antibodies against mouse CD45, CD8, CD44, and CD62L. Shown are the gating (**a**) and plots of one mouse from each treatment group (**b**).

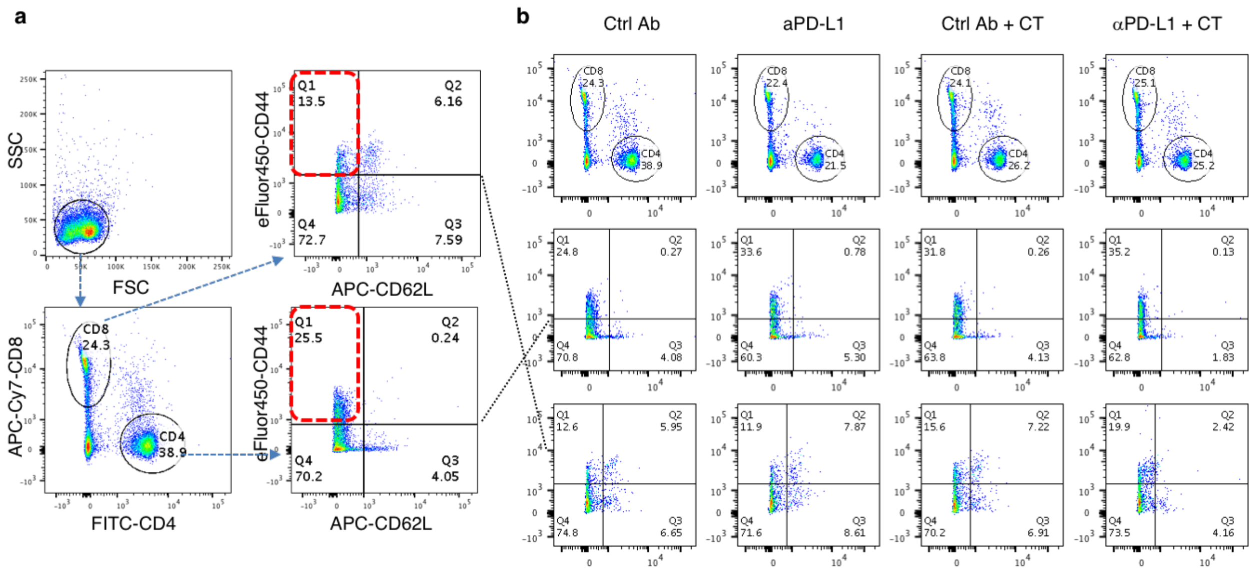

sFig. 5. Flow cytometry measurement of effector/memory (CD62L⁻/CD44^{high}) CD4 and CD8 T cells in the tumor draining lymph nodes of treated Hepa1-6-bearing mice. Draining lymph nodes (dLN) were removed from Hepa1-6-bearing C57BL/6 mice 24 h after the 3rd treatment with CT (100 μg/mouse) and anti-PD-L1 (αPD-L1, 10 μg/mouse) alone or in combination and immunostained with labeled antibodies against mouse CD4, CD8, CD44, and CD62L. Shown are the gating (**a**) and plots of one mouse from each treatment group (**b**).

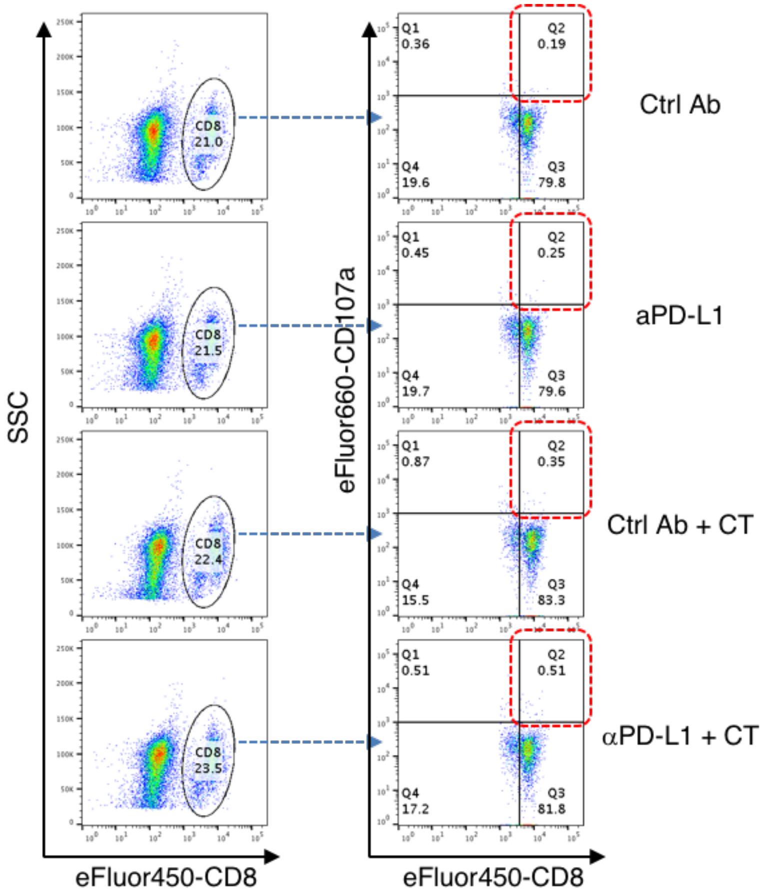

sFig. 6. Functional CTLs in the draining lymph nodes of treated Hepa1-6-bearing mice. Draining lymph nodes (dLN) removed from Hepa1-6-bearing C57BL/6 mice 24 h after the 3rd treatment with CT (100 μg/mouse) and anti-PD-L1 (αPD-L1, 10 μg/mouse) alone or in combination were used to make single cell suspension. Subsequently, dLN cells were seeded into a monolayer of Hepa1-6 in a 48-well plate at a ratio of 20:1 (dLN cell:Hepa1-6), cocultured for 18 h in a CO_2 incubator, harvested, stained with eFluor450-CD8 and eFluor660-CD107a, and analyzed by flow cytometry. Shown are the gating and plots of one mouse of each treatment group.

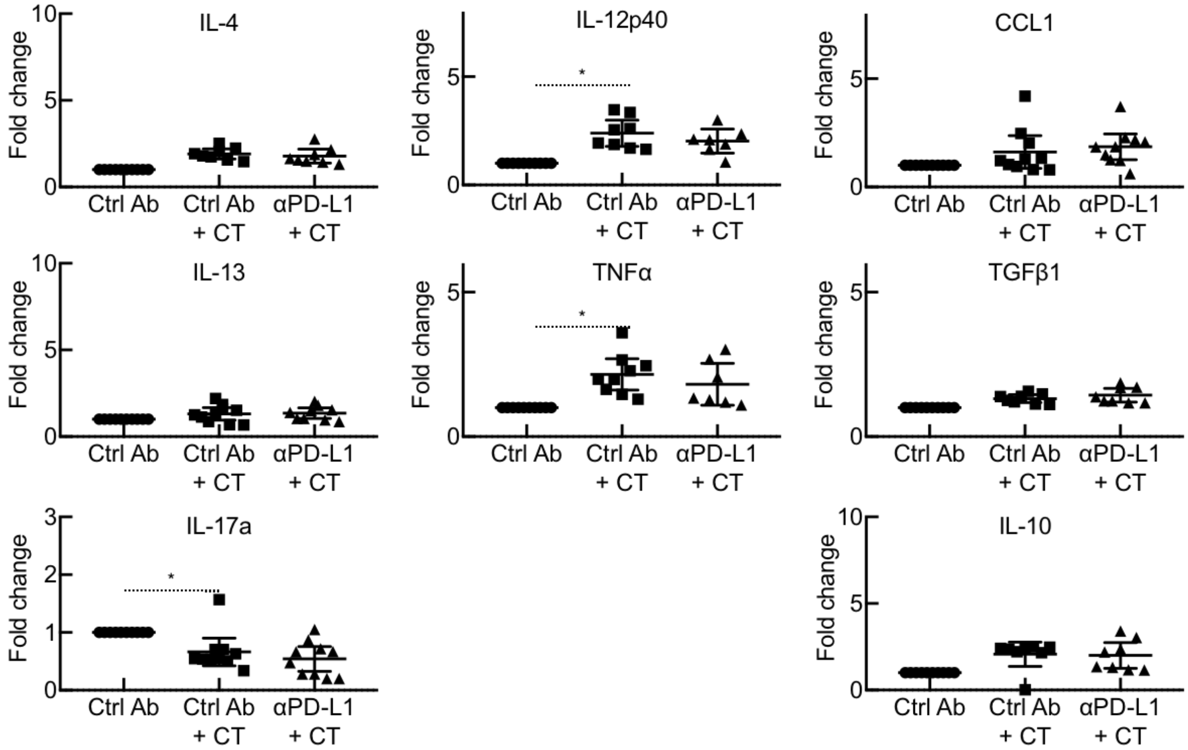

sFig. 7. Cytokines expressed in Hepa1-6 tumors in response to treatment. RNAs were extracted from Hepa1-6 tumors resected 24 h after the 3rd treatment with CT (100 μg/mouse) and anti-PD-L1 (αPD-L1, 10 μg/mouse) alone or in combination. The expression of target genes was measured by qPCR, analyzed using Qiagen website, and presented as fold change. *p<0.001 (n = 7~10).

sTable 1. qPCR primers used in the current study

Primer Name	Company	Primer Number
Mouse CXCL9	Qiagen	PPM029723-200
Mouse CXCL10	Qiagen	PPM02978E-200
Mouse CXCL11	Qiagen	PPM03192C-200
Mouse CCL1	Qiagen	PPM03138C-200
Mouse IFNα11	Qiagen	PPM03050B-200
Mouse IFNβ	Qiagen	PPMO3594C-200
Mouse TGFβ1	Qiagen	PPM02991B-200
Mouse Perforin	Qiagen	PPM34456B-200
Mouse Granzyme B	Qiagen	PPM05303F-200
Mouse IFNγ	Qiagen	PPM03121A-200
Mouse IL-17a	Qiagen	PPM03023A-200
Mouse iNOS	IDT	199797838
Mouse IL-4	Qiagen	PPM03013F-200
Mouse IL-10	Qiagen	PPM03017C-200
Mouse IL-13	IDT	135048668
Mouse TNFα	IDT	155731475
Mouse IL-12p40	IDT	155731469
Mouse GAPDH	IDT	135048676
